# Breathing Maneuvers as a Vasoactive Stimulus for Detecting Inducible Myocardial Ischemia – An Experimental Cardiovascular Magnetic Resonance Study

**DOI:** 10.1371/journal.pone.0164524

**Published:** 2016-10-14

**Authors:** Kady Fischer, Dominik P Guensch, Nancy Shie, Julie Lebel, Matthias G Friedrich

**Affiliations:** 1 Philippa & Marvin Carsley CMR Centre at the Montreal Heart Institute, Université de Montréal, Montreal, QC, Canada; 2 University Hospital Bern, Department Anaesthesiology and Pain Therapy, Inselspital, University of Bern, Bern, Switzerland; 3 University Hospital Bern, Institute for Diagnostic, Interventional and Paediatric Radiology, Inselspital, University of Bern, Bern, Switzerland; 4 Research Institute of the McGill University Health Centre, Montreal, QC, Canada; 5 Department of Radiology, Université de Montréal, Montreal, QC, Canada; 6 Departments of Medicine and Diagnostic Radiology, McGill University, Montreal, QC, Canada; 7 Department of Cardiology, Heidelberg University Hospital, Heidelberg, Germany; 8 Departments of Cardiac Sciences and Radiology, University of Calgary, Calgary, Canada; Universitatsklinikum Wurzburg, GERMANY

## Abstract

**Background:**

Breathing maneuvers can elicit a similar vascular response as vasodilatory agents like adenosine; yet, their potential diagnostic utility in the presence of coronary artery stenosis is unknown. The objective of the study is to investigate if breathing maneuvers can non-invasively detect inducible ischemia in an experimental animal model when the myocardium is imaged with oxygenation-sensitive cardiovascular magnetic resonance (OS-CMR).

**Methods and Findings:**

In 11 anesthetised swine with experimentally induced significant stenosis (fractional flow reserve <0.75) of the left anterior descending coronary artery (LAD) and 9 control animals, OS-CMR at 3T was performed during two different breathing maneuvers, a long breath-hold; and a combined maneuver of 60s of hyperventilation followed by a long breath-hold. The resulting change of myocardial oxygenation was compared to the invasive measurements of coronary blood flow, blood gases, and oxygen extraction. In control animals, all breathing maneuvers could significantly alter coronary blood flow as hyperventilation decreased coronary blood flow by 34±23%. A long breath-hold alone led to an increase of 97±88%, while the increase was 346±327% (*p*<0.001), when the long breath-hold was performed after hyperventilation. In stenosis animals, the coronary blood flow response was attenuated after both hyperventilation and the following breath-hold. This was matched by the observed oxygenation response as breath-holds following hyperventilation consistently yielded a significant difference in the signal of the MRI images between the perfusion territory of the stenosis LAD and remote myocardium. There was no difference between the coronary territories during the other breathing maneuvers or in the control group at any point.

**Conclusion:**

In an experimental animal model, the response to a combined breathing maneuver of hyperventilation with subsequent breath-holding is blunted in myocardium subject to significant coronary artery stenosis. This maneuver may allow for detecting severe coronary artery stenosis and have a significant clinical potential as a non-pharmacological method for diagnostic testing in patients with suspected coronary artery disease.

## Introduction

The numbers for prescribed cardiac diagnostic tests and interventions are rapidly growing[1]. Notably, imaging for inducible myocardial ischemia or coronary artery stenosis has become one of the most critical cost factors in today’s health care systems[2]. While imaging techniques such as stress echocardiography and nuclear cardiology are useful for identifying ischemia-producing coronary artery stenosis, they require pharmacological or physical stress protocols. Nuclear techniques are also limited by radioactivity of tracers. Cardiovascular magnetic resonance imaging (CMR) can identify significant coronary artery stenosis without radiation, commonly using either dobutamine stress or first-pass perfusion protocols[3,4]. Yet again, the infusion of pharmacological stress agents or contrast media is required, increasing effort, cost and procedure-related risk.

Oxygenation-sensitive CMR (OS-CMR) imaging allows for monitoring changes of myocardial oxygenation, based on the so-called blood oxygen level-dependent (BOLD) effect: A reduction of tissue oxygenation leads to a relative increase of deoxyhemoglobin, which in turn causes a signal intensity (SI) drop in CMR images sensitive to this effect[5]. In the presence of coronary artery stenosis, this effect can be augmented further by post-stenotic capillary recruitment[6]. In addition to the lack of radiation and contrast agents, OS-CMR differs from perfusion imaging techniques in that it directly reflects the oxygenation status of the tissue instead of using surrogate markers such as blood supply.

Alterations of blood gases by breathing maneuvers or inhalation of gas mixtures were identified as an alternative to pharmacologic vasodilation in functional MRI studies of the brain and more recently the heart[7–9]. In particular, a combination of hyperventilation and breath-holds with OS-CMR yielded promising results in monitoring changes of myocardial oxygenation induced by vasoactive maneuvers[10–12], and could induce significant changes in myocardial perfusion as detected by first pass perfusion CMR[13]. Yet, its clinical potential to identify an abnormal regional response of the coronary vasculature in the presence of severe coronary artery stenosis has not been explored.

We hypothesized that the vascular and thus oxygenation response to breathing maneuvers, especially a long breath-hold following hyperventilation, is blunted in myocardium exposed to severe coronary artery stenosis.

## Methods

### Animal Preparation

This study was conducted in accordance with the Guide to the Care and Use of Experimental Animals by the Canadian Council on Animal Care and approved by the local Animal Care and Use Board of the Montreal Heart Institute [#20125002]. Twenty healthy swine (33±1kg, Yorkshire-Landrace) were included. The animals were pre-medicated with 4ml Telazol (200mg tiletamine, 200mg zolazepam, i.m.) and 0.8mg atropine. Anesthesia was induced with propofol (2-4mg/kg, i.v.) and after intubation, maintained with continuous propofol (4–36 mg/kg/hr, i.v.) and remifentanil (0-3.5μg/kg/min, i.v.) infusion as required. Amiodarone (75mg i.v.) was infused, and serum electrolytes were corrected to normal values to prevent arrhythmia[14]. The femoral vein was cannulated for drug and fluid administration, while the femoral artery was used for hemodynamic monitoring and arterial blood gas analyses. A right jugular vein sheath was placed to provide access to the coronary sinus for venous blood gas analyses. Through left-sided thoracotomy, an MR-compatible perivascular flow probe was implanted onto the proximal left anterior descending (LAD) coronary artery (Transonic Systems, Ithica, NY, USA). Nine animals served as controls, while eleven animals were allocated to undergo a LAD stenosis protocol. After instrumentation, a bolus of 5000U heparin was administered to prevent clotting.

### Stenosis Protocol

**Fig 1:**
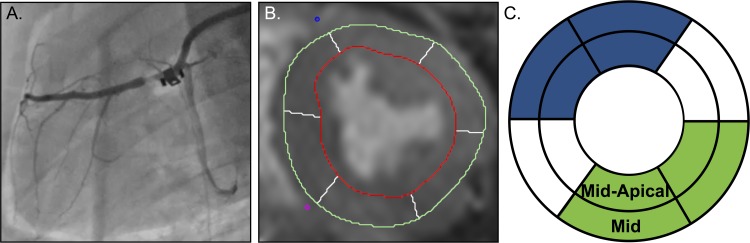
Experimental setup. A. Fluoroscopy image of the left anterior descending (LAD) coronary artery with the perivascular occluder the flow probe in place. B. Example of OS-CMR image with AHA automatic segmentation. C. Assignment of segments to LAD territory (blue) and remote myocardium (green), excluding the segments with possible mixed perfusion.

A perivascular hydraulic occluder (In Vivo Metric, CA, USA) was used to constrict the LAD adjacent to the flow probe (Fig 1). Vasodilation was induced with 140μg/kg/min adenosine administered through a central vein. FFR was measured with a coronary pressure guidewire (St. Jude Medical, St Paul, MN, USA). An occluder was inflated to constrict the vessel until a stable FFR reading of <0.75 was reached. This occlusion was maintained throughout the study. In control animals, an FFR of 1.0 was assumed.

### Quantitative Coronary Angiography

Quantitative coronary angiography (QCA) was performed prior to CMR imaging using a standard protocol[15]. Minimal LAD diameters were measured in the occluder-obstructed area, for the stenosis group, or distal to the flow probe for the control animals using the online single-plane view imaging system (model 1.2.3; Electromed International, QC, Canada).

### Experimental Protocol

**Fig 2:**
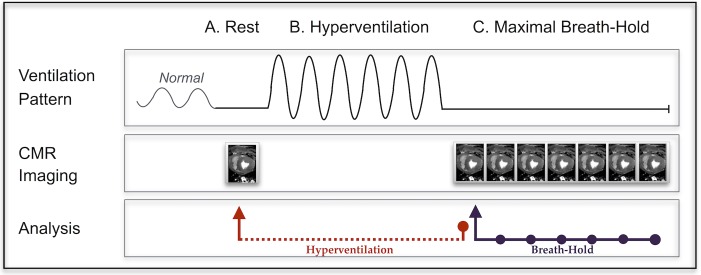
Breathing maneuver protocol. For the combined hyperventilation breath-hold (HVBH) maneuver, a single rest measurement was obtained in a short breath-hold (A). The animal was then manually hyperventilated for 60s (B) followed immediately by a long breath-hold (C) that was imaged throughout, with a repeating OS sequence. Hyperventilation analysis was always compared between rest and the start of the breath-hold (red arrow), while the breath-hold could be analyzed at multiple time points with comparison to data obtained at the beginning of the breath-hold (purple arrow). The long breath-hold (LBH) followed step C, starting after a normal ventilation pattern.

Animals were transferred to an MRI suite and unless specified otherwise, arterial blood gases were maintained at a baseline normoxic (paO_2_ = 100mmHg) and normocapnic (paCO_2_ = 40mmHg) levels, targeted through ventilation adjustments. CMR images were acquired with a clinical 3T MRI system (MAGNETOM Skyra 3T; Siemens Healthcare, Erlangen, Germany) using an 18-channel cardiac phased array coil. All images were obtained during breath-holds, created by interrupting ventilation at end-expiration, after a passive exhalation. Left ventricular function was imaged with a standard ECG-gated balanced steady-state free precession (SSFP) cine in a short-axis stack (echo time 1.43ms, repetition time 3.3ms, flip angle 65°, voxel size 1.6x1.6x6.0mm, matrix 192x120, bandwidth 962Hz/Px). Then, OS-CMR imaging was performed for two short axis slices, mid-ventricular and mid-apical distal to the blood flow probe, using a previously published ECG triggered SSFP sequence (echo time 1.70ms, repetition time 3.4ms, flip angle 35°, voxel size 2.0x2.0x10.0mm, matrix 192x120, bandwidth 1302Hz/Px)[16] with the following two breathing maneuvers:

- HV/HVBH: Combined hyperventilation and breath-hold. After the rest OS-CMR images were obtained. animals were manually hyperventilated (HV) for 60s at a rate of 30–40 breaths/min with a breathing bag and an additional supplement of 2-4L/min of oxygen to maintain normoxia. This was immediately followed with a 60-90s breath-hold (HVBH). Images were acquired continuously during breath-holds (Fig 2).
- LBH: long breath-hold. A breath-hold of 60s was performed from the baseline level and imaged continuously.

At the beginning and end of each maneuver, the following invasive measurements were recorded: coronary blood flow, arterial and coronary sinus blood gases, heart-rate, and invasive blood pressure. The myocardial oxygen extraction ratio (O_2_er) was calculated from the oxygen content of the arterial (CaO_2_) and coronary sinus blood (CcsO_2_), [O_2_er = (CaO_2_-CcsO_2_) / CaO_2_]. For the breath-holds (LBH & HVBH), all measurements were compared to the data obtained at the beginning of the breath-hold to measure the change only within the breath-hold. Hyperventilation-induced changes were analyzed as a separate component and the measurements obtained at pre-hyperventilation were used as the baseline for this maneuver. After complete data acquisition, 200mg propofol and 40mmolKCl (i.v.) were administered for euthanasia.

### Image Analysis

All CMR images were de-identified prior to analysis to blind the reader from the degree of stenosis. Signal intensity was measured using certified CMR image evaluation software (cvi^42^, Circle Cardiovascular Imaging, Calgary, AB, Canada). The myocardial oxygenation response from the end-systolic OS-CMR images was expressed as the % signal intensity change (ΔSI[%]) compared to the baseline for each measurement. For each group, ΔSI[%] of myocardium of the LAD territory, i.e. antero-septal and anterior segments in mid-ventricular and mid-apical slices was averaged and compared with that of inferior and inferolateral remote myocardium (Fig 1C). The segments from the inferoseptal and the anterolateral wall were not included in the direct comparisons due to a known possible mixed perfusion pattern[17]. Segments were entirely excluded if more than 33% of the segment area was removed during analysis due to artifact.

### Statistical Analysis

Data is expressed as mean±SD. Continuous variables were assessed for normal distribution with the D’Agostino-Pearson test. Paired t-tests were used to compare the changes in variables from baseline within an animal, while independent t-tests compared data between groups. If both analyses were needed, a two-way mixed ANOVA with multiple comparisons analysis was performed. Specifically, for the OS-CMR data, the ΔSI[%] response of the LAD region in stenotic animals was compared to the remote tissue to determine a regional abnormality, and to the control animals at the same time point using a mixed two-way model. Associations between ΔSI[%] of the LAD region of all animals and FFR, O_2_er, and coronary flow were assessed with Pearson’s correlation. The breath-holds were further visualized by plotting the ΔSI[%] over time, fitted by a least-squares non-linear regression. For assessing inter-observer reliability, 54 randomly selected OS-images from 10 animals were read by an independent second reader and assessed with a two-way mixed intraclass-correlation test. Tests were performed with GraphPad Prism version 6.0 for mac (GraphPad Software, La Jolla California USA) and SPSS (IBM SPSS Statistics for Mac, Version 21.0, New York, USA: IBM Corp). Results were considered statistically significant with a two-tailed *p*<0.05.

## Results

Eight control animals successfully completed all maneuvers; one death occurred during surgery. In the stenosis group, one death occurred during the induction of coronary artery stenosis, resulting in 10 subjects. Anesthetics were individually adjusted to the requirements of each animal. After surgery, a higher average dose and range of remifentanil was required for control animals than the stenosis animals (0.26±0.42 (0 to 1.28) vs. 0.07±0.17 (0 to 0.55) μg/kg/min, *p* = 0.009), with an equal dose of propofol (19.4±4.7 (15.5 to 33.0) vs. 16.5±4.3 (9.5 to 22.0) mg/kg/hr, *p* = 0.198).

### Fractional Flow Reserve, Quantitative Angiography and Coronary Flow

All induced coronary artery stenoses were significant, based on QCA measurements (Table 1, S1 Data) and hemodynamically significant, with a mean FFR of 0.63±0.05 (range 0.54–0.74). The control animals had insignificant LAD diameter reductions (7±7%).

**Table 1:** Baseline Function and Angiography.

Parameter	Control (n = 8)	Stenosis (n = 10)	p
**O_2_er (%)**	47±8	67±13	0.001
**EF (%)**	54±11	48±10	0.262
**CO (L/min)**	2.8±0.8	2.1±0.5	0.041
**HR (beats/min)**	93±14	84±18	0.282
**Coronary artery stenosis**			
QCA, %-Diameter	7±7	63±11	<0.001
QCA, %-Surface Area	11±8	85±6	<0.001
FFR	N/A	0.63±0.05	

Mean±SD values of the baseline myocardial oxygen extraction ratio (O_2_er), ejection fraction (EF), cardiac output (CO), heart rate (HR), the %-decrease in diameter and surface area obtained from quantitative coronary angiography (QCA), and the fractional flow reserve value (FFR).

**Fig 3:**
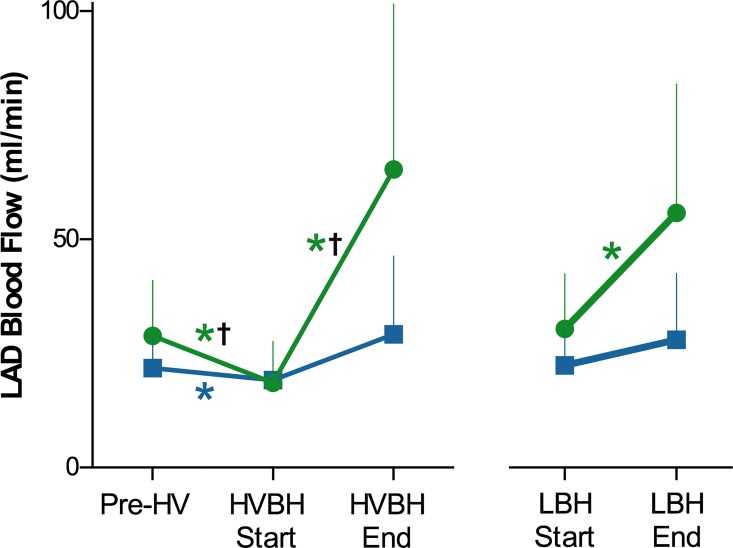
Changes of coronary blood flow during breathing maneuvers. Mean changes of coronary blood flow (ml/min) induced by breathing maneuvers in control (green) and stenosis (blue) animals. All maneuvers significantly changed the flow (*p<0.05) for control animals, while only hyperventilation altered the coronary blood flow for the stenosis group, (%-change is listed in S1 Table). Both hyperventilation (HV) and the hyperventilation breath-hold (HVBH) triggered a flow response that was significantly different between stenosis and control animals (†*p*<0.05), while there was no statistically significant difference between the groups observed with the long breath-hold (LBH).

In control animals, all breathing maneuvers had a statistically significant impact on coronary blood flow (Fig 3); hyperventilation decreased coronary blood flow by 34±23% (29±12 to 18±9 ml/min, *p*<0.001), while breath-holds had vasodilating properties: LBH increased blood flow by 97±88% (30±12 to 56±28ml/min, *p* = 0.005 vs baseline), and HVBH had an even stronger response of 346±327% (18±9 to 65±35ml/min, *p* = 0.001 vs baseline). For the stenosed group, HV and HVBH were the only maneuvers to show an attenuated flow response when compared with control animals (p<0.001, S1 Table). During hyperventilation, the stenosed animals also had a decrease of 12±6% in coronary flow from the baseline (22±8 to 19±7ml/min (p = 0.032), however, this was a smaller change than that observed in control animals (p<0.001). In this group, the breath-holds did not yield a significant change in coronary blood flow compared to baseline (HVBH: +82±110% to 29±17ml/min, p = 0.278; LBH: 40±60% from 22±8 to 28±15ml/min, p = 0.083).

### Ventricular Function

The stenosis group had a slightly lower ejection fraction and lower cardiac output, but this difference was only significant for cardiac output (Table 1). Baseline heart rate was not significantly different between the groups.

### Blood Gases and Oxygen Extraction Ratio

Breathing maneuvers significantly affected arterial and coronary sinus blood gas values (S1 Table). While hyperventilation decreased pCO_2_ and increased pO_2_, an opposite effect was induced by the breath-holds, HVBH and LBH. Most changes did not statistically differ between groups. In animals with coronary artery stenosis however, there was a 22±11% greater drop in the arterial hemoglobin saturation (SaO_2_) than in control animals (*p* = 0.014) during HVBH and a 25±11% greater drop during the LBH (*p* = 0.005). Furthermore, while baseline O_2_er was significantly lower in the control than in stenosis animals (Table 1), relative changes in O_2_er during each maneuver from these baselines did not significantly differ between groups.

### OS-CMR Image Quality

Image quality was generally good and only 4.2% of segments had to be excluded from the analysis (2.7% and 5.7% of mid and mid-apical slice of segments, respectively). Agreement between the readers was acceptable (ICC: 0.80, 95%CI: 0.66–0.89).

### Myocardial Oxygenation

**Fig 4:**
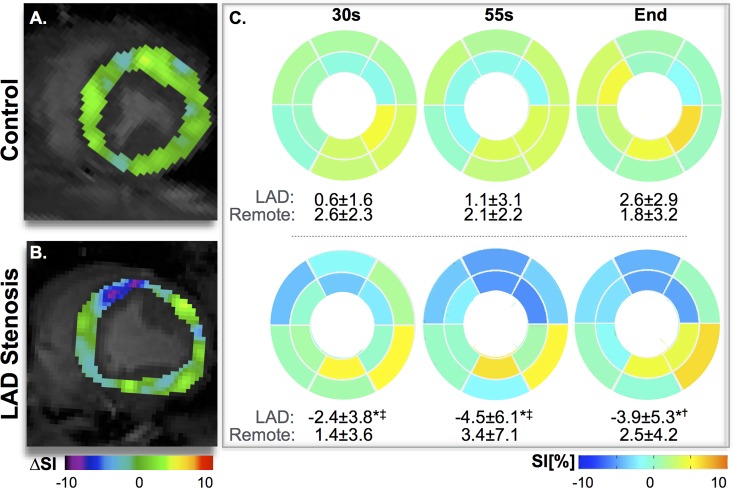
Segmental changes of myocardial oxygenation during the HVBH. Subtraction images (smoothed using a 6mm Gaussian filter) demonstrate that at the 30s, SI increased homogeneously in the control animal (A), while there was a decrease in the territory of the stenosed LAD (B). The mean response for each segment from all animals similarly shows that in control animals (top row, n = 8), ΔSI[%] is consistently larger for all segments, whereas for the stenosis animals (bottom row, n = 10) in the LAD regions a significant decrease is already observed at 30s, and this continues throughout the breath-hold. (*p<0.05 between LAD and remote territory within the group, †*p*<0.05, <0.05‡*p*<0.01 for the difference in LAD response between groups).

**Fig 5:**
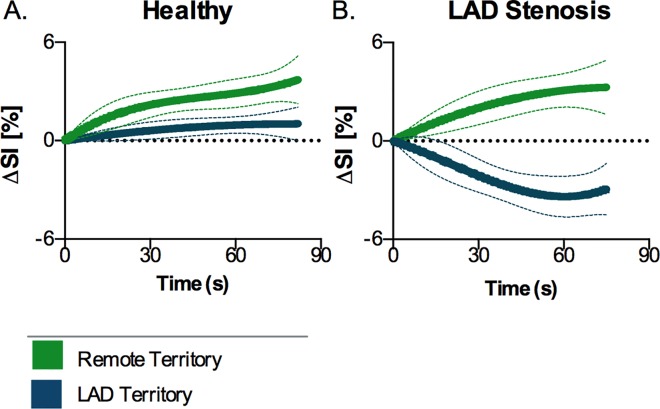
Myocardial oxygenation response curve during the HVBH. Signal intensity increases globally during the HVBH in control animals (A), yet the animals with a stenosis (B) show a significant decrease in the LAD territory (blue), while the remote region (green) remains above baseline with a similar characteristic of the control animals.

A consistent significant reduction in signal (ΔSI[%]) was observed in the LAD region with the HVBH in stenosis animals (Fig 4). Hyperventilation alone did not induce any significant changes in the SI, (healthy controls: LAD -0.2±2.6%; remote -2.3±4.4%, and stenosis group: LAD -0.7±2.6%; remote -2.8±6.1%). Thirty seconds into the breath-hold after hyperventilation, the SI of the LAD region had already dropped by 2.4±3.8% in the presence of stenosis (Fig 5). The response in this region was significantly lower (*p* = 0.001) than in remote myocardium, and was non-significantly lower than the same region of the control animals (*p* = 0.090). In remote myocardium, SI change was minimal (1.4±3.6%), and comparable to both regions of control animals (LAD: +0.6±1.6%, remote: +2.6±2.3%). The LAD signal of the stenosis animals continued to decrease throughout the breath-hold ending at -3.9±5.3 below baseline, while all other regions remained above baseline (*p* = 0.001). For the long breath-hold maneuver without preceding hyperventilation (LBH), the SI in the LAD segments in the stenosis animals was not significantly different from the control LAD region at 30s (-1.9±1.0% vs 0.5±2.1%, p = 0.100) or at the end of the maneuver (-2.9±2.9% vs -0.7±6.0%). Furthermore in this maneuver, regional abnormalities were not observed.

### Relation to FFR and Other Measurements

**Fig 6:**
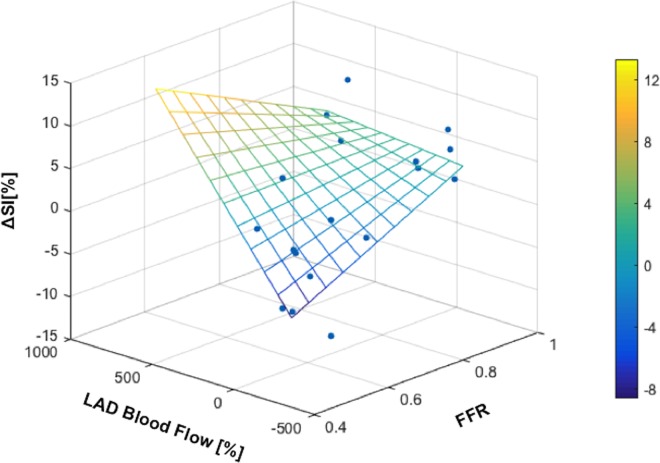
Myocardial oxygenation in relation to coronary blood flow and FFR. SI at the end of the HVBH is linearly related to both the coronary blood flow response (%) and the FFR (Table 2).

The ΔSI[%] in the LAD region of the HVBH was well correlated with the FFR at all time-points in the breath-hold (Table 2). HVBH was the only maneuver that was closely related to the change in coronary blood flow (Fig 6). During the LBH, the SI at 30s was correlated to FFR. No significant relationships were observed with oxygenation responses during hyperventilation.

**Table 2:** Correlation of the regional oxygenation response to invasive measurements.

Dependent Variable ΔSI[%]-LAD	Independent Variables	Correlation Coefficient	p
**HV**	FFR	0.056	0.826
	Flow	-0.153	0.543
	O_2_er	-0.107	0.120
**HVBH**			
**30s**	FFR	0.461	0.040
**55s**	FFR	0.478	0.043
**End**	FFR	0.604	0.009
	Flow	0.465	0.040
	O_2_er	0.237	0.360
**LBH**			
**30s**	FFR	0.655	0.003
**End**	FFR	0.145	0.288
	Flow	0.045	0.864
	O_2_er	0.167	0.521

Correlation (n = 18) analyses were performed for all breathing manoeuvres with the oxygenation response (ΔSI[%]) of the left anterior descending (LAD) perfused territory as the dependent variable. All time points were compared to the fractional flow reserve (FFR), and to changes in LAD flow, oxygen extraction ratio (O_2_er).

## Discussion

Our results indicate that the regional oxygenation response to a combined breathing maneuver of hyperventilation followed by apnea is significantly altered in the presence of an acute coronary artery stenosis. This breathing maneuver may therefore have potential as a non-pharmacological vasodilator for diagnostic purposes.

Currently, the administration of pharmacologic agents such as adenosine is considered the standard procedure for assessing vascular function by inducing vasodilatation. We could recently show that in healthy humans, the HVBH maneuver may yield greater changes in myocardial oxygenation than standard peripheral iv infusion of adenosine[16], which is expected to induce near-maximal coronary vasodilatation[18]. In this experimental animal study, we found that the combination of OS-CMR with a combined breathing maneuver (HVBH) consistently revealed regional myocardial oxygenation abnormalities caused by significant coronary artery stenosis.

For this technique, we have chosen to use balanced SSFP sequences for the OS imaging. The signal in this type of sequence is proportional to (M0*T2/T1), and the output signal is already dependent on the signal decay sensitive to hemoglobin de-oxygenation, without requiring more advanced signal processing. While this method may not be as sensitive as directly measuring signal relaxation times, it is advantageous as these sequences are faster, widely available in all MRI scanners, and this simpler analysis could aid the adoption of the technique clinically.

### Impact on Coronary Blood Flow and Myocardial Oxygenation

The data are consistent with and extend the current understanding of coronary vascular physiology. An altered change of regional myocardial oxygenation due to a blunted coronary vascular response to pharmacological vasodilation has been reported[19]. Breathing maneuvers appear suitable to assess vascular reactivity because of their impact on blood gas levels: Apnea leads to hypercapnia and hypoxia, which trigger vasodilation, while hyperventilation with associated hypocapnia and hyperoxia induce vasoconstriction[20,21]. By using a combined breathing maneuver, mild hypocapnia can be induced by hyperventilation, allowing for a greater range of CO_2_ changes and better tolerance of the subsequent breath-hold[22,23] and thus a larger change of the vascular tone. A clear distinction in the coronary flow response between control and stenosis animals was observed with the HV and following HVBH breathing maneuver. This was paralleled by marked regional differences of myocardial oxygenation during the HVBH maneuver between hypoperfused and remote myocardium. Furthermore, this was matched by a healthy volunteer study which showed the same breathing maneuver could induce a change in myocardial blood flow, as assessed by first pass perfusion CMR[13].

### Comparison Between Breathing Maneuvers and Current Techniques

Standard adenosine protocols provide binary snapshots between baseline and a hyperemic state. Breathing maneuvers on the other hand allow for monitoring dynamic changes over time and thus provide additional information with respect to the dynamics of a vascular response. As a potential disadvantage, changes of the saturation of incoming blood and of the cardiac workload during the maneuvers could be confounders. Of note, breathing patterns may also affect other current myocardial perfusion techniques[13], for example with varying durations of breath-holds during CMR or CT scans or hyperventilation in anxious patients during cardiology tests.

As the signal intensity in the OS images is dependent on the local dHb fraction, there are competing effects during the breathing maneuvers. The breath-holds are primarily a vasodilating stimulus. In theory, the oxygen supply and demand balance is uncoupled and there is a “luxury” perfusion of the myocardium reducing the regional dHb fraction, and thus increasing the OS-CMR signal. However, as the breath-hold continues, arterial blood can become globally desaturated, causing a lesser increase in signal or even a reduction at the end of the breath-hold as indicated by the curve in Fig 5. In the presence of vascular dysfunction, we expect that the vasodilating component of the breath-holds will not be as competitive in the compromised arteries and there will be an attenuated signal intensity response. This can even lead to signal intensity decrease in the presence of coronary steal, because of the increased vascular resistance from the fixed stenosis. On the other hand, hyperventilation is a known vasoconstrictor that does not significantly affect the arterial blood saturation. Thus, if the reduction in blood supply is not matched by reduction in oxygen demand, myocardial oxygenation should theoretically be reduced[16].

Different from previous human studies[12,16], hyperventilation did not induce a significant change in myocardial oxygenation, yet there was a significant decrease in coronary blood flow, and increase in blood oxygen content in both groups. The reduced decrease in SI compared to healthy volunteers may be a direct effect of anaesthesia, known to reduce oxygen requirements throughout the body. Therefore, the drop in blood flow may not have had a smaller impact on the myocardium in these anaesthetized swine with less oxygen extracted than in awake subjects. Probably related to the preceding hyperventilation with vasoconstriction, there was a more pronounced response during a subsequent breath-hold (HVBH) when compared with a long breath-hold (LBH) alone. Consequently, HVBH data were also more consistent than LBH results with respect to identifying regional abnormalities caused by coronary artery stenosis. The marked decrease in SI in the territory subtended by the stenotic coronary artery could be explained by a possible delayed recovery from the vasoconstrictive stimulus of hyperventilation, in combination with a coronary steal phenomenon caused by the competing vasodilation in remote myocardium[24,25] and post-stenotic capillary recruitment that results in a pooling of deoxygenated blood[6]. Thus the HVBH maneuver may exploit two mechanisms associated with an abnormal vascular response in a hypoperfused coronary territory. This is corroborated by a previous human study in healthy participants, where HVBH had a much stronger effect on the oxygenation response than the LBH[16].

Breathing maneuvers also have an impact on blood oxygenation, which is a key determinant in the OS-CMR signal. It is likely that during the breath-holds, there is luxury perfusion due to the hypercapnia, which could explain the early SI increase in the breath-hold observed in healthy animals. Only with continued apnea, net blood oxygenation and subsequently tissue oxygenation drops[26]. In the presence of coronary artery stenosis however, the combination of hypoxemia and hypercapnia may actually induce or worsen myocardial ischemia in the jeopardized territory. In fact, we observed that in the presence of severe coronary artery stenosis, the lack of a proper compensatory increase in myocardial blood flow allows an immediate decrease in tissue oxygenation in the tissue subtended to the stenosis, in contrast to an SI increase in both, remote tissue and in control animals (Fig 5). This pattern was correlated with both, the functional severity of the stenosis (FFR) and with the observed change in coronary blood flow.

For control animals, the relatively small but insignificant difference between the LAD and remote territories may also reflect physiologic variations between coronary territories. Other studies have demonstrated that in healthy subjects, the anterior wall often had a more pronounced perfusion or oxygenation response than other territories[17,27,28].

### Limitations and Future Directions

In the present animal study we observed a weaker response to hyperventilation, including a smaller change of SI and heart rate in comparison with previous reports in humans. This may be caused by the anesthesia through its known cardio-depressant effects and suppression of vegetative reflexes. This interference could theoretically attenuate the vascular response to endogenous vasodilators. Further, anaesthesia is known to block vegetative reflexes, such as sympathetic activation during hypercapnia, which may have an impact on blood flow or heart rate as well.

The breath-holds were maintained despite significant changes in blood gases levels, more than would be tolerated by conscious human subjects[29]. Healthy human participants are able to comfortably undergo similarly long breath-holds, but with lesser changes in blood gases[12], thus these results may not translate directly to human responses. Cardiac patients may not tolerate such long breath-holds, but with both, volunteers and the current animal model, significant CMR results were observed within the first 30s of the breathing maneuvers, allowing for clinically feasible protocols[16]. In a pilot study, we have already successfully applied the HVBH maneuver in patients with obstructive sleep apnea syndrome, who all tolerated the procedure well, with the majority maintaining a breath-hold after hyperventilation of more than 30s[30], at which point an oxygenation difference could be observed between the groups as well.

Our animals did have an acute regional stenosis with otherwise presumably healthy microvasculature. Differences in compensatory mechanisms in more chronic forms may alter the vascular response and it remains to be studied, whether the exact values we obtained can be directly translated to humans. Additionally, the LAD coronary flow measurements did not monitor the response of the other two coronary arteries and furthermore did not precisely measure coronary flow reserves as tissue perfusion is regulated primarily by microvascular resistance, and not by the large conduit vessels[31]. Similarly, the coronary sinus collects blood from the other coronary arteries as well, which dilutes the oxygen extraction calculations from the LAD territory. Thus these measurements illustrate some partial effects, but do not completely describe the coronary flow and oxygen extraction responses of the entire heart.

## Conclusion

Breathing maneuvers have a profound effect on coronary blood flow and myocardial oxygenation. Specifically, hyperventilation with subsequent breath-holding leads to a significant vasoactive response, which is markedly compromised in myocardium subtended by a significantly stenotic coronary artery. The proposed combination of oxygenation-sensitive CMR with breathing maneuvers appears to be useful for diagnostic testing in patients with suspected coronary artery stenosis, especially when contrast agents or stress protocols are not suitable. Further studies are now warranted to investigate its clinical utility in patients with suspected myocardial ischemia.

## Supporting Information

S1 Data Raw Data. Data of each subject (n = 18) for the reported values are provided in Microsoft Excel format.
(XLSX)

S1 Table Invasive Measurements. Mean±SD change in values during the breathing maneuvers (* denotes significance (p<0.05) from baseline value). † O_2_er baseline values were systematically higher in the stenosis animals as compared to control animals as shown in Table 1. Arterial partial pressure of carbon dioxide (paCO_2_); coronary sinus partial pressure of carbon dioxide (pcsCO_2_); arterial partial pressure of oxygen (paO_2_); coronary sinus partial pressure of oxygen (pcsO_2_); arterial saturation (SaO_2_); oxygen extraction ratio (O_2_er); heart rate (HR); and rate pressure product (RPP).
(DOCX)
