# Supplementary material for: Breathing Maneuvers as a Vasoactive Stimulus for Detecting Inducible Myocardial Ischemia – An Experimental Cardiovascular Magnetic Resonance Study
Source: PLoS One. 2016 Oct 14;11(10):e0164524. doi: 10.1371/journal.pone.0164524 (PMC5065132; doi:10.1371/journal.pone.0164524)
Supplement: S1 Table — Mean±SD change in values during the breathing maneuvers (* denotes significance (p<0.05) from baseline value). † O2er baseline values were systematically higher in the stenosis animals as compared to control animals as shown in Table 1. Arterial partial pressure of carbon dioxide (paCO2); coronary sinus partial pressure of carbon dioxide (pcsCO2); arterial partial pressure of oxygen (paO2); coronary sinus partial pressure of oxygen (pcsO2); arterial saturation (SaO2); oxygen extraction ratio (O2er); heart rate (HR); and rate pressure product (RPP). (DOCX) [file pone.0164524.s002.docx]

**Supplementary Table 1: Invasive Measurements**

| Parameter | Control | Stenosed | P |
| --- | --- | --- | --- |
| %-change Flow |  |  |  |
| HV | -34 ± 23* | -12 ± 6* | <0.001 |
| HVBH | 346 ± 327* | 82 ±110 | 0.004 |
| LBH | 97 ± 88* | 40 ± 60 | 0.066 |
| ΔpaCO_2_  (mmHg) |  |  |  |
| HV | -15 ± 5* | -15 ± 3* | 0.887 |
| HVBH | 27± 9* | 20 ± 8* | 0.046 |
| LBH | 15 ± 5* | 12 ± 5* | 0.250 |
| ΔpcsCO_2_ (mmHg) |  |  |  |
| HV | -3 ± 2* | -8 ± 5* | <0.001 |
| HVBH | 6 ± 6* | 10 ± 3* | 0.094 |
| LBH | 10 ± 7* | 9 ± 5* | 0.842 |
| ΔpaO_2_ (mmHg) |  |  |  |
| HV | 73 ± 71* | 70 ± 30* | 0.975 |
| HVBH | -117 ± 48* | -135 ± 30* | 0.315 |
| LBH | -73 ± 9* | -80 ± 12* | 0.104 |
| ΔpcsO_2_ (mmHg) |  |  |  |
| HV | 0 ± 2 | 1 ± 2 | 0.136 |
| HVBH | -12 ± 7* | -13 ± 7* | 0.961 |
| LBH | -13 ± 7* | -15 ± 7* | 0.802 |
| ΔSaO_2_ (%) |  |  |  |
| HV | 0 ± 0 | 0 ± 0 | - |
| HVBH | -30 ± 23* | -52 ± 24* | 0.014 |
| LBH | -36 ± 30* | -61 ± 15* | 0.005 |
| ΔO_2_er (%)† |  |  |  |
| HV | -2 ± 6 | -4 ± 7 | 0.639 |
| HVBH | 20 ± 20* | 2 ± 28 | 0.091 |
| LBH | 16 ± 24 | 10 ± 16 | 0.678 |
| ΔHR (bpm) |  |  |  |
| HV | 3 ± 11 | 0 ± 8 | 0.798 |
| HVBH | 3 ± 14 | 2± 12 | 0.996 |
| LBH | 8 ± 20 | -1 ± 14 | 0.208 |
| ΔRPP (mmHg*bpm) |  |  |  |
| HV | -2311±2141* | -712±2006 | 0.055 |
| HVBH | -560±1285 | -1459±5580 | 0.106 |
| LBH | -4617±3292* | -2961±2835* | 0.216 |

Mean±SD change in values during the breathing maneuvers (* denotes significance (p<0.05) from baseline value). † O_2_er baseline values were systematically higher in the stenosed animals as compared to control animals as shown in Table 1. Arterial partial pressure of carbon dioxide (paCO_2_); coronary sinus partial pressure of carbon dioxide (pcsCO_2_); arterial partial pressure of oxygen (paO_2_); coronary sinus partial pressure of oxygen (pcsO_2_); arterial saturation (SaO_2_); oxygen extraction ratio (O_2_er); heart rate (HR); and rate pressure product (RPP).
